# Spatio-Temporal Structure, Path Characteristics, and Perceptual Grouping in Immediate Serial Spatial Recall

**DOI:** 10.3389/fpsyg.2016.01686

**Published:** 2016-11-11

**Authors:** Carlo De Lillo, Melissa Kirby, Daniel Poole

**Affiliations:** Department of Neuroscience, Psychology and Behaviour, University of LeicesterLeicester, UK

**Keywords:** Corsi test, grouping, spatial span, serial recall, spatial memory, working memory, chunking, virtual reality

## Abstract

Immediate serial spatial recall measures the ability to retain sequences of locations in short-term memory and is considered the spatial equivalent of digit span. It is tested by requiring participants to reproduce sequences of movements performed by an experimenter or displayed on a monitor. Different organizational factors dramatically affect serial spatial recall but they are often confounded or underspecified. Untangling them is crucial for the characterization of working-memory models and for establishing the contribution of structure and memory capacity to spatial span. We report five experiments assessing the relative role and independence of factors that have been reported in the literature. Experiment 1 disentangled the effects of spatial clustering and path-length by manipulating the distance of items displayed on a touchscreen monitor. Long-path sequences segregated by spatial clusters were compared with short-path sequences not segregated by clusters. Recall was more accurate for sequences segregated by clusters independently from path-length. Experiment 2 featured conditions where temporal pauses were introduced between or within cluster boundaries during the presentation of sequences with the same paths. Thus, the temporal structure of the sequences was either consistent or inconsistent with a hierarchical representation based on segmentation by spatial clusters but the effect of structure could not be confounded with effects of path-characteristics. Pauses at cluster boundaries yielded more accurate recall, as predicted by a hierarchical model. In Experiment 3, the systematic manipulation of sequence structure, path-length, and presence of path-crossings of sequences showed that structure explained most of the variance, followed by the presence/absence of path-crossings, and path-length. Experiments 4 and 5 replicated the results of the previous experiments in immersive virtual reality navigation tasks where the viewpoint of the observer changed dynamically during encoding and recall. This suggested that the effects of structure in spatial span are not dependent on perceptual grouping processes induced by the aerial view of the stimulus array typically afforded by spatial recall tasks. These results demonstrate the independence of coding strategies based on structure from effects of path characteristics and perceptual grouping in immediate serial spatial recall.

## Introduction

One of the most enduring problems in psychology and the neurosciences is the characterization of the mechanisms supporting the representation of serial order information (Lashley, 1951; Rosenbaum et al., 2007; Hurlstone et al., 2014). Serial Spatial Recall (SSR) refers to the ability to temporarily retain a sequence of spatial locations in a prescribed order and is one of the most common instantiations of the problem of serial order in short-term and working memory. The assessment of SSR is of central importance in several areas of psychological research. It has been used to evaluate the extent to which the processing of serial order in the verbal and visuo-spatial domain rests on similar mechanisms (Baddeley, 1992; Smyth and Scholey, 1992; Jones et al., 1995; Hurlstone et al., 2014), a crucial issue for the characterization of human cognitive architecture. SSR is one of the most widespread neuropsychological measures (Berch et al., 1998; Kessels et al., 2000) and is included as a test in widely used batteries (e.g., WAIS-R, Kaplan et al., 1991; Wechsler, 1997a; Wechsler Memory Scale, WMS-III, Wechsler, 1997b; Cantab, Cambridge Cognition, 2006). SSR has been extensively employed in the study of individual differences in working-memory (Cornoldi and Vecchi, 2003) and as a predictor of scholastic achievement (Jarvis and Gathercole, 2003; St Clair-Thompson, 2007). Because of its non-verbal nature, the assessment of SSR has been used for the comparison of memory skills in monkeys and humans, with important implications for the evaluation of primate models of human memory (Botvinick et al., 2009; Fagot and De Lillo, 2011).

Despite the popularity of SSR as a psychological measure and its suitability for addressing the problem of serial order from a cognitive, comparative, and neuropsychological perspective, its cognitive bases are still poorly understood and, as argued below, a number of central constructs for its description are often confounded. One of the most important issues to address in relation to SSR, as identified by a recent eminent review (Hurlstone et al., 2014) and as further elaborated below, is the characterization of the organizational factors that can contribute to accurate SSR (e.g., Kemps, 1999, 2001; Bor et al., 2003; De Lillo, 2004; Busch et al., 2005; Parmentier et al., 2005; Rossi-Arnaud et al., 2005; Parmentier and Andrés, 2006; Parmentier et al., 2006; Ridgeway, 2006; Imbo et al., 2009; De Lillo and Lesk, 2010).

SSR is typically measured by assessing spatial span with the Corsi test (Milner, 1971; Corsi, 1972), allegedly the most widely used non-verbal neuropsychological test (Berch et al., 1998; Kessels et al., 2000). In the Corsi test participants observe a sequence of spatial items, such as a series of finger tapping movements across an array of wooden blocks, or a series of flashing icons presented on a touch-screen. Then, they are required to reproduce the series by tapping the items in the same order. Because the items are all identical in shape and color, they need to be identified by their spatial position. For this reason, the Corsi test is considered one of the purest measures of spatial memory span (see Baddeley, 2001, for a review).

Traditionally, the Corsi test has featured irregular arrays of items and random sequences as recall material (Milner, 1971). However, it was realized soon that that not all random sequences are recalled at the same level of accuracy (Smirni et al., 1983) and attempts to standardize the test ensued with important applied implications for the use of these tests for clinical diagnosis (Kessels et al., 2000; Busch et al., 2005).

The complexity of Corsi sequences has been manipulated in order to assess the relative autonomy of short and long-term memory structures (Kemps, 1999). The results of ingenious experiments have clarified that items in spatial working-memory are coded configurationally, using allocentric frames of reference (Avons, 2007; Avons and Oswald, 2008; Boduroglu and Shah, 2014), thus highlighting the role of relational properties of items in the display in recall.

Some studies (De Lillo, 2004, 2012; De Lillo and Lesk, 2010) have emphasized the notion that the understanding of the effects of organizational factors in SSR is of interest apart from the assessment of memory span *per se*. They proposed that with the irregular spatial arrangement of the items in Corsi-type tasks and randomly selected sequences of block tapping (see Berch et al., 1998 for examples of Corsi displays and criteria for selecting sequences that have been used in the literature), it is impossible to isolate the effect of particular organizational factors and interpret them in relation to the memory representation that they afford.

In order to assess the contribution of a specific type of organizational factor on spatial span De Lillo (2004) used a Corsi display, presented on a touch-screen, where 9 squares were arranged spatially to form 3 clusters of 3 items each, so that the separation of the items within clusters was inferior to that between clusters. The use of a configuration of items grouped in spatial clusters was motivated by different considerations. It seemed the appropriate way to convey in a Corsi task the fact that space can be divided in different sub-regions. It provided a spatial analogy of forms of semantic clustering and chunking observed in non-spatial domains. Finally, a configuration of items grouped in spatial clusters resembles a “patchy” foraging environment that according to foraging theories of cognitive evolution provided the pressures for the emergence of large brain and working memory skills in humans and other primates (e.g., Milton, 1993). Importantly, the use of a clustered Corsi display with items arranged in spatial clusters enables the manipulation of the serial organization of sequences so that they can be made either compatible or not with chunking by spatial proximity. De Lillo (2004) used different types of sequences. Some sequences were segregated by clusters, so that consecutive items were always in the same cluster and a transition to a different cluster occurred only after all the items within a cluster had been selected. Clustered sequences were deemed to afford a hierarchical representation because the order of the clusters, into which the sequence was segregated, could be stored independently from the order of the items within a given cluster. Other sequences were designed to be incompatible with such hierarchical organization because consecutive items were always in different clusters.

When recall for the two types of sequences was compared a beneficial “clustering effect” emerged; sequences segregated by clusters were reported at a higher level of accuracy. Consistently with a hierarchical model, in sequences segregated by clusters, longer Response Times (RTs) emerged at cluster boundaries. This suggested that the retention of spatially clustered sequences could be supported by a hierarchical representation similar to that observed for chunking in non-spatial domains (Miller, 1956; Klahr et al., 1983). By contrast, non-clustered sequences showed longer reaction times for the items at intermediate ordinal positions within the sequences. This is a pattern of RT that resembles the serial position curve typically observed for lists of unrelated items in other domains, such as nonsense words, where items at intermediate ordinal positions are the most difficult to recall.

The recall of Corsi sequences typically shows a long initial RT which is indicative of the processing of serial order just before recall (see Fischer, 2001). Further evidence for the hierarchical representation of clustered Corsi sequences has been provided by showing that a component of this initial RT is proportional to the number of clusters in which sequences are segregated and RTs at cluster boundaries that are proportional to the number of items within each cluster (De Lillo and Lesk, 2010).

The neural correlates of the processing of organizational factors in SSR have been highlighted in an f-MRI study (Bor et al., 2003) where participants faced an array of items arranged as a square matrix and were presented with “structured” and “unstructured” sequences. “Structured” sequences were operationally defined as those sequences where consecutive items were within the same row, column or diagonal. “Unstructured” sequences were defined as those violating this constraint (i.e., with consecutive items never within the same row, column, or diagonal). Behavioral results confirmed that structured sequences were reported at a higher level of accuracy than unstructured sequences. Moreover, f-MRI data indicated a higher activation of the dorsolateral prefrontal cortex (DLPFC) during the encoding of structured sequences than during the encoding of unstructured sequences.

Other important factors have been reported to affect the reproduction of spatial sequences. These include the length of the path of the trajectory necessary to connect all the items in the sequence and the number of times the path crosses itself (Orsini et al., 2001, 2004; Parmentier et al., 2005, 2006). These factors sometimes confound the effects of the structure of the representation underpinning performance. For example, it has been proposed that the clustering effect as observed by De Lillo (2004) could be explained by the fact that clustered sequences can have on average a shorter path length than non-clustered sequences (Parmentier et al., 2006). Similarly, the effect of structure observed by Bor et al. (2003) could be due to the fact that unstructured sequences can contain more crossings.

The RT patterns reported for structured sequences (Bor et al., 2003; De Lillo, 2004; De Lillo and Lesk, 2010) and the fMRI results of Bor et al. (2003) suggest that the detection and use of structure in Corsi sequences determines the formation of specific forms of hierarchical representation that contribute to efficient recall quite apart from other effects of path characteristics. Nevertheless, considering the possible contribution of all these factors, it is important to assess their relative role in SSR. We attempted to do so with the present study. The approach we took in the first experiment was to dissociate path-length and organization in a clustered array. With this experiment we tested the notion proposed by Parmentier et al. (2006) that path-length can be the sole explanation of the clustering effect in spatial span. We manipulated display size so that clustered sequences in a large display had a longer path-length than non-clustered sequences in a small display. Thus, if the benefits of clustering are explained by the shorter path that is normally associated with clustered sequences, then we should expect a more accurate recall for the non-clustered sequences with a short path when compared with the recall for structured sequences with a longer path. In the second experiment, we manipulated the timing structure of the sequence leaving its path-length and any other characteristics of the sequences unchanged. Using the same clustered sequences, we imposed pauses in the sequence presentation either at transitions between items within a cluster or at cluster boundary. An effect of timing in this experiment would indicate that the clustering effect is more likely to be related to the way in which the sequence is represented, rather than to mere effects of path characteristics, such as path-length or number of path-crossings.

In a third experiment we used a square matrix of locations which allowed a fully factorial manipulation of path length, presence of crossings, and structure as defined by Bor et al. (2003). By doing so we aimed to disentangle the effects of path length, presence of crossings, and structure. We then determined which of these factors explained most of the variance in the recall score of the participants.

The aim of the fourth and fifth experiments was to evaluate the importance of perceptual grouping for the emergence of beneficial effects of structure in SSR. In fact, the use of terms such as perceptual grouping, perceptual organization and gestalt principles is so widespread in the literature in relation to the explanation of the benefits of organizational factors in SSR and so often used interchangeably with that of efficient memory coding (e.g., Kemps, 1999; Bor et al., 2003; Rossi-Arnaud et al., 2005; Ridgeway, 2006; Bor, 2012; Hurlstone et al., 2014) to warrant an explicit assessment of the extent to which perceptual grouping is actually required for the benefits of organization in SSR to emerge.

In Experiments 4 and 5 we used immersive virtual reality to implement a navigational version of the Corsi task. In this task the order in which the sequence items had to be reproduced could not be apprehended from the same viewpoint. Having identified the first item in the sequence participants were required to move toward it and select it. Only then would the next item be presented at a different location within the environment. The presentation of the sequence was a lengthy process that involved continuous changes of directions and viewpoints. We reasoned that the observation of beneficial effects of structure in these conditions would have made the hypothesis that perceptual grouping processes are necessary for their emergence implausible.

## Experiment 1

In SSR, path length refers to the length of the trajectory that connects the items that need to be reported in the prescribed order. It can be manipulated independently from other characteristics of the spatial sequences by altering the size of the item display so that the relative distance between the items is different in the two displays (Smyth and Scholey, 1994). The critical variable in this experiment was the relative distance of the items in the small and the large display. The experiment was designed so that clustered sequences, presented in the large set, had a longer path-length than non-clustered sequences presented in the small set. If the short path that typically accompanies clustered sequences is the sole explanation of the clustering effect, as proposed by Parmentier et al. (2006), then non-clustered sequences with a shorter path should be recalled more accurately than clustered sequences with a longer path.

### Methods

#### Participants

Twenty five volunteers (10 male and 15 females), with a mean age of 26 years (SD = 7.76), were recruited from a participant panel at the University of Leicester and paid a small fee to take part in the experiment. They all reported normal or corrected-to-normal vision.

#### Materials, design, and procedure

**Figure 1:**
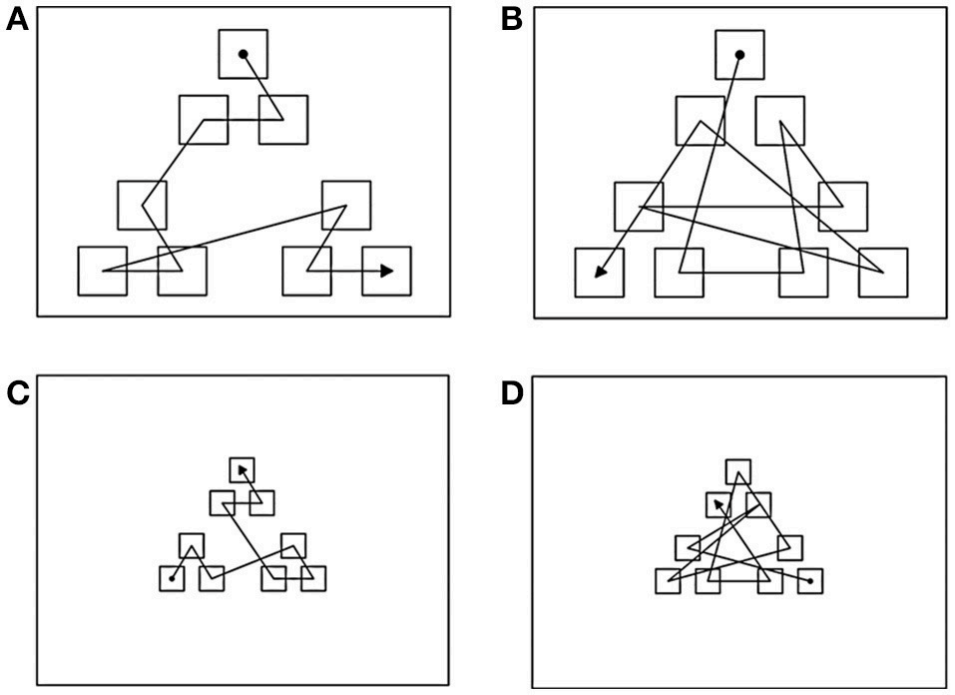
**The layout of the display used for experiment 1 and examples of sequence paths for conditions: (A)** Long structured; **(B)** Long unstructured; **(C)** Short structured; and **(D)** Short unstructured. Filled circle indicates the start of the sequence and arrow point indicates the ending position. The lines indicate the order in which the icons “blinked” during the presentation phase and were not actually displayed. See text for explanation.

The experiment was presented using a PC equipped with a 17″ Elo, IntelliTouch sensitive monitor (1024 × 768 pixels). A visual display consisting of a black background and nine identical gray squares arranged in three spatial clusters of three icons each was presented on each trial. Two displays were used: a large display, composed of squares 120 pixels wide (Figures 1A,B); and a small display, composed of squares 40 pixels wide (Figures 1C,D). In the small display, there was a 6 pixel-wide invisible active border area surrounding each square to ensure that the touch of a square was accurately registered even by participants with larger finger tips.

In the large display, squares within a cluster were separated by a distance between 76 and 103 pixels, whereas the distance between clusters was of 152–189 pixels. In the small display, squares within a cluster had a distance between 19 and 27 pixels and the distance between clusters was of 38–53 pixels.

In each trial, participants were first presented with the full display of 9 squares for 700 ms. One square then turned to black for 500 ms, before the full display was represented for another 700 ms. This produced the impression that the icon would “blink.” Another square would then turn black for 500 ms, and so on until a sequence of 9 items was presented in this way. After the ninth square had blinked, the screen turned black for 1 s. The full display was then presented again and participants were required to reproduce the sequence that they had previously observed. To confirm that the touch had been registered, each square turned to black for 50 ms when touched.

The design featured the manipulation of sequence type that could be either “Structured” or “Unstructured” and path-length that could be “Long” or “Short.”

“Structured” sequences were segregated by spatial clusters, so that all the items of each cluster were presented before the sequence moved to a different cluster. By contrast “Unstructured” sequences were not segregated by clusters, so that consecutive items were always presented in different clusters. “Long” sequences were displayed in the large display and “Short” sequences were presented in the small display. The average path-length of long sequences was of 3093.36 pixels (SE = 170.37) and that of short sequences was 883.69 pixels (SE = 50.14).

Four experimental conditions were obtained combining these two factors in a 2 (structure) × 2 (path-length) repeated measures design: Long Structured (L-S), Long Unstructured (L-U), Short Structured (S-S), and Short Unstructured (S-U). Importantly, the path-length of L-S sequences (mean = 2381.32; SE = 40.09) was significantly longer than that of S-U sequences (mean = 1096.32; SE = 17.42). Examples of each sequence type are also provided in Figure 1. Ten sequences of each type were used and presented in random order within a testing session of 40 trials.

Participants were tested in a quiet laboratory with dim lighting. The height of their chair was adjusted so their eyes were at the same level as the center of the screen and they could comfortably touch any point of the display with the index finger of their dominant hand. Participants were informed that they had to use that finger when selecting the squares during the experiment, which took about 15 min to complete.

This experiment and all the other experiments reported in this article were carried out in accordance with the Code of Ethics and Conduct of the British Psychological Society and approved by the University of Leicester Ethics Committee for research involving human participants (Psychology sub-committee). All subjects gave written informed consent in accordance with the Declaration of Helsinki.

### Results

#### Accuracy

**Figure 2:**
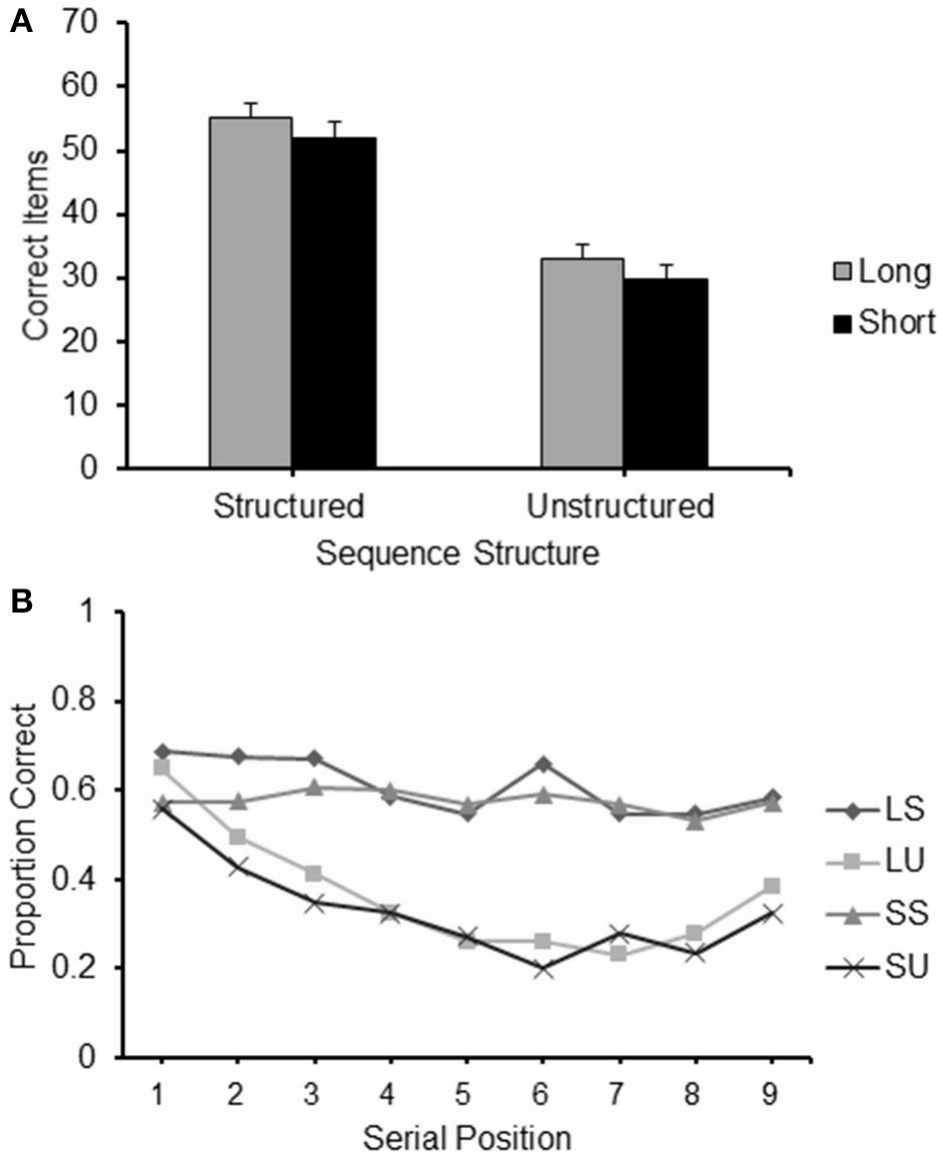
**(A)** Frequency of items correct recalled for the different conditions (Long Structured; Short Structured; Long Unstructured and Short Unstructured) of Experiment 1; **(B)** Proportion of correct items recalled at each serial position for the four different conditions of Experiment 1: LS, Long Structured; LU, Long Unstructured; SS, Short Structured; SU, Short Unstructured.

An item recalled correctly was defined as a square touched in the correct serial position. Accuracy scores were the frequency of items correctly recalled by each participant in each condition. The mean accuracy score of each of the four conditions is presented in Figure 2A.

A 2 (structure: structured/unstructured) × 2 (path-length: long/short) repeated measures ANOVA was carried out on the frequency of correct items reported in the different conditions. It revealed a significant main effect for structure [*F*_(1, 24)_ = 8.747, *p* < 0.01, $\eta_{\text{p}}^{2}$ = 0.267] with a higher level of recall for structured sequences and path-length [*F*_(1, 24)_ = 198.965, *p* < 0.001, $\eta_{\text{p}}^{2}$ = 0.892], with a higher level of recall for long sequences. No interaction between path-length and structure was found.

Paired sample *t*-tests with Bonferroni correction (alpha of 0.05 corrected to 0.01 and alpha of 0.01 corrected to 0.002) were carried out to further clarify these results. Long structured sequences produced a significantly higher level of accuracy on recall than short unstructured sequences [*t*_(24)_ = 12.404, *p* < 0.01], demonstrating that structured sequences were recalled at a higher level of accuracy that unstructured sequences even when they had a longer path-length. Moreover, the effect of structure was very robust as it was maintained in both short [*t*_(24)_ = 11.681, *p* < 0.01] and long sequences [*t*_(24)_ = 11.208, *p* < 0.01]. The effect of path-length proved less robust as it did not emerge when sequences with long and short path-length were compared within the structured and unstructured conditions separately.

#### Serial position analysis

Serial position effects were observed in each condition, as can be observed for the serial position curves presented in Figure 2B. A 2 (path-length: long/short) × 2 (structure: structured/unstructured) × 9 (serial position: 1/2/3/4/5/6/7/8/9) ANOVA for repeated measures was carried out. A significant main effect emerged for path-length [*F*_(1, 24)_ = 8.747, *p* < 0.01, $\eta_{\text{p}}^{2}$ = 0.267], structure [*F*_(1, 24)_ = 198.965, *p* < 0.001, $\eta_{\text{p}}^{2}$ = 0.892], and serial position [*F*_(8, 192)_ = 26.326, *p* < 0.001, $\eta_{\text{p}}^{2}$ = 0.523], confirming the main results reported above. A particularly strong interaction emerged between structure and serial position [*F*_(8, 192)_ = 13.082, *p* < 0.001, η^2^p = 0.345]. As can be observed from Figure 2B this can be easily accounted for by the different shape of the curves of the structured sequences on the one hand, and the unstructured sequences on the other. The latter curves resemble typical serial position curves for unstructured material with some indications of possible primacy and recency effects (see Crowder, 1969). Such effects are absent in the structured sequences. A significant interaction between path-length and serial position was also found, [*F*_(8, 192)_ = 5.510, *p* < 0.01, $\eta_{\text{p}}^{2}\;=$ 0.114]. This was not as conspicuous as the interaction between serial position and cluster type. It is likely to be explained by small differences occurring at different serial positions, which are more difficult to pinpoint. The third order interaction between path-length, structure and serial position was not significant.

### Discussion

In this experiment we observed beneficial effects of clustering similar to those observed in other studies (De Lillo, 2004; De Lillo and Lesk, 2010). We found that clustering had a beneficial effect in both sequences with a long and with a short path. This suggests that path-length alone is unlikely to explain the effects of clustering in SSR. It has previously been suggested that clustered sequences afford a hierarchical coding of the sequence with spatial clusters forming the superordinate level and the items within each cluster forming the subordinate level (e.g., De Lillo, 2004; De Lillo and Lesk, 2010). However, Experiment 1 was not designed to assess this possibility. An attempt at gaining a better insight on the type of memory coding supported by clustering in this study was made in Experiment 2 by manipulating the temporal pattern of the presentation of clustered sequences.

## Experiment 2

Experiment 2 aimed to provide additional support for the independence of the effects of structure and path-length in SSR and some indication concerning the nature of the representation underlying sequences segregated by spatial clusters. In order to do so, we used an approach previously used in the study of chunking and hierarchical representation in recall in the spatial (Bor et al., 2003) and other domains (Farrell and Lelievre, 2012). The temporal structure of the presentation of the sequences was manipulated by inserting temporal pauses during the presentation of clustered sequences. Only clustered sequences were used in this experiment. For some sequences the pause was inserted at transitions between items within a cluster. For other sequences, the pause was inserted at transitions between clusters. As such, sequences could be either consistent or inconsistent with a hierarchical representation based on segmentation by spatial clusters. Other path characteristics of the sequences remained the same so that effects of the temporal structure of the sequence could not be confounded with other effects of path characteristics.

### Methods

#### Participants

Twenty five undergraduate psychology students (23 females and 2 males, age range 18–25 years) from the University of Leicester took part in this experiment as part of their course requirement. All participants reported normal or corrected-to-normal vision.

#### Materials, design, and procedure

**Figure 3:**
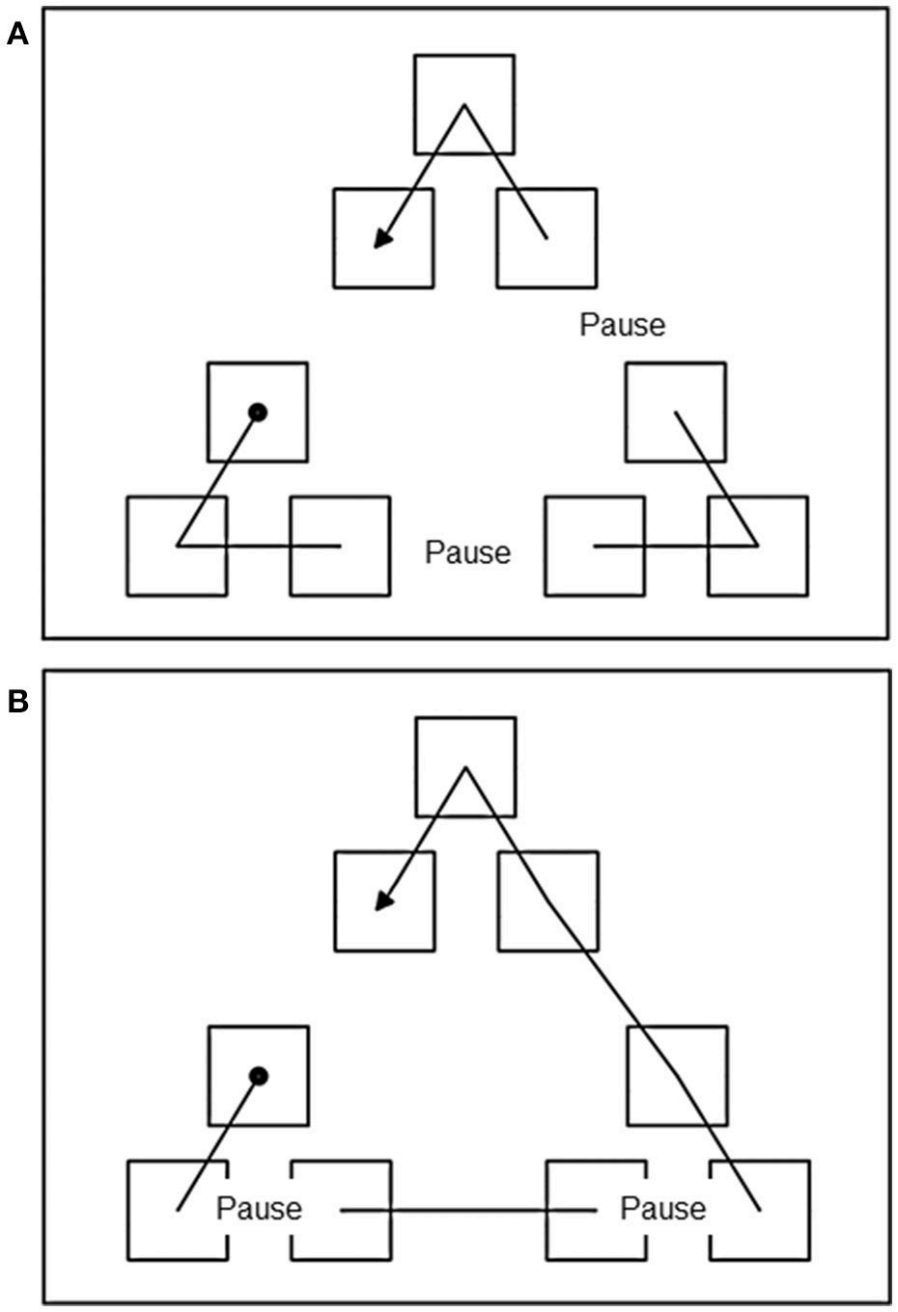
**(A)** Example of a trial of the pause between cluster condition of Experiment 2. Dashed lines represent the position of the pause. Filled circle indicates the start of the sequence and arrow point indicates the ending position. **(B)** Example of a trial of the pause within cluster condition of Experiment 2. Dashed lines represent the position of the pause. Filled circle indicates the start of the sequence and arrow point indicates the ending position. The lines indicate the order in which the icons “blinked” during the presentation phase and were not actually displayed. See text for explanation.

The same PC used in Experiment 1 was used here. The static display was the same as the large display of Experiment 1. The presentation of sequences followed the same general procedure of Experiment1, with the following exception. In Experiment 2, a 3 s pause was introduced at critical points during the presentation of the sequence according to different conditions as described below. To discourage the participants from fixating the last item that blinked before the pause, thus minimizing any effects of the temporal pauses, during the pauses all the items disappeared (turned the same color of the background). In the Between Cluster Pause (BCP) condition, the pause was inserted when the sequence reached a cluster boundary and a transition between clusters was required. In the Within Clusters Pause (WCP) condition, the pause was inserted at a transition between items in the same cluster (see Figure 3 for a visual representation of a BCP and a WCP sequence, illustrating this procedure).

Eighteen different sequences were used. Each sequence was displayed twice, once as part of the BCP condition and once as part of the WCP condition. Two pauses were presented in each sequence of both conditions.

Thus, a total of 36 sequences were presented to each participant in random order. As exactly the same sequences were presented for the BCP and of the WCP condition, we ensured that the path length and any other path characteristic were the same in the sequences of both conditions.

### Results

**Figure 4:**
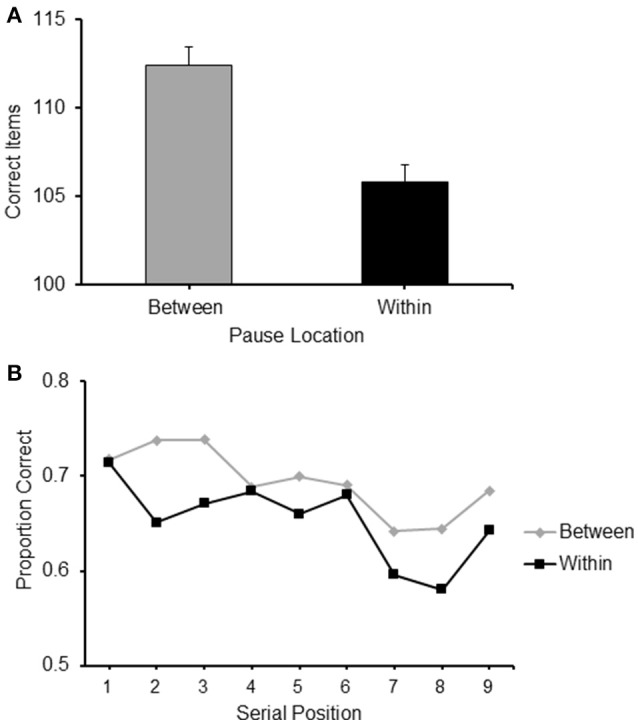
**(A)** Frequency of items correct recalled for the two conditions of Experiment 2 where the pauses location during sequence presentation was Between or Within clusters, respectively. See text for explanation. **(B)** Proportion of correct items recalled at each serial position for the two conditions of Experiment 2: Pauses Between and Pauses Within clusters.

The mean frequency of items correctly recalled for sequences with BCP and WCP are presented in Figure 4A. It can be observed from there that BCP sequences were recalled more accurately than WCP sequences.

A paired-sample *t*-test confirmed that this difference was significant [*t*_(24)_ = 2.727, *p* < 0.05].

#### Serial position analysis

A 2 (pause location: between/within) × 9 (serial position: 1/2/3/4/5/6/7/8/9) repeated measure ANOVA carried out on the proportion of items correctly recalled confirmed an effect of pause location [*F*_(1, 24)_ = 7.334, *p* < 0.05, $\eta_{\text{p}}^{2}=$ 0.234] and revealed a significant effect of serial position [*F*_(8, 192)_ = 5.007, *p* < 0.001, $\eta_{\text{p}}^{2}=$ 0.173] (see Figure 4B).

### Discussion

Experiment 2 clarifies the possible causes of the beneficial effects of spatial clustering in SSR. The pattern of results of this experiment is consistent with the pattern that is usually considered evidence for chunking in other domains. During the presentation of the to-be-recalled material, pauses at the predicted locations of chunking boundaries (which were compatible with the formation of a hierarchical representation based on grouping by spatial proximity) produced a higher level of recall accuracy than pauses imposed within the predicted chunk boundaries (which presumably hindered the hierarchical organization of the sequence based on spatial proximity). Moreover, in this experiment exactly the same sequences, and as such the same sequence paths, were used for BCP and for the WCP. Only the temporal pattern of the presentation of the sequences was manipulated. Therefore, the effects observed here cannot be accounted for by factors related to the path characteristics of the sequence such as path-length or number of crossings featured in a given sequence (Orsini et al., 2001, 2004; Parmentier et al., 2005; Parmentier and Andrés, 2006).

Taken together, the results of the first two experiments support the notion that participants use spatio-temporal structure in immediate serial recall tasks to form representations, such as a hierarchical representation of the sequence based on spatial clusters, to enhance recall (De Lillo, 2004; De Lillo and Lesk, 2010).

In Experiments 1 and 2 we used a clustered arrangement of items. The use of such an arrangement is important for several reasons. It provides a spatial analogy of class formation in other domains. It conforms to the most intuitive hierarchical organization of space, which is in regions and sub-regions. Moreover, the study of recall of clustered locations is particularly relevant in relation to the potential role played by the pressure to search efficiently patchy resources in the evolution of sophisticated memory skills in humans and other primates as advocated by foraging theories of primate cognitive evolution (Milton, 1993).

Nevertheless, the use of spatially clustered items does not offer the flexibility necessary for the manipulation of path-length and number of path-crossings in sequences which are either structured or unstructured, in a fully nested factorial design. Therefore, in order to explore further the relative role of effects of structure and effects of different path characteristics, in Experiment 3 we used a large matrix of locations which allowed such experimental design.

## Experiment 3

Sequence structure (Bor et al., 2003; De Lillo, 2004; De Lillo and Lesk, 2010; Fagot and De Lillo, 2011) and the path characteristics of sequences have been shown to affect SSR (Kemps, 2001; Orsini et al., 2001, 2004; Parmentier et al., 2005; Parmentier and Andrés, 2006; Fagot and De Lillo, 2011). Among path characteristics, the presence of path-crossings in the sequence has proved an important variable that can substantially affect recall. Path-crossings refers to the occasions where the imaginary line traced by connecting all the items in the sequence in the prescribed order, crosses itself. Whereas, effects of path-length on SSR have failed to emerge in some studies on human participants (Smyth and Scholey, 1994; Fagot and De Lillo, 2011), negative effects of the presence of path-crossings have emerged consistently in studies where this variable has been manipulated (Orsini et al., 2001, 2004; Parmentier et al., 2005; Parmentier and Andrés, 2006; Fagot and De Lillo, 2011). Path-length and path-crossings are often confounded because sequences containing more crossings will on average be longer than sequences with less crossings. Thus, some studies have used the strategy of keeping sequence length constant when assessing the effect of path crossing (Parmentier and Andrés, 2006). However, the relative contribution of path-length, path-crossings, and any other possible residual effects of structure that cannot be explained by these two path characteristics has not been evaluated yet in a single factorial experiment. We aimed to do so in experiment 3. Clustered arrays, such as those used in Experiments 1 and 2 offered us the opportunity to study effects of hierarchical organization based on spatial proximity on SSR. Another form of organization that has been reported to have a beneficial effect on SSR is operationally defined using arrays of items arranged as a 4 × 4 square matrix of locations (Bor et al., 2003). Structured sequences, defined as those where consecutive items are within the same row, column or diagonal are recalled with a higher level of accuracy than sequences that violate this rule. These beneficial effects of structure have been considered to be related to chunking (Bor et al., 2003). However, here too effects of structure, path-length and crossings can be confounded. A systematic assessment of the relative role of structure and path characteristics within these arrays has not been attempted yet, possibly also because a 4 × 4 grid is not large enough to allow the generation of sequences that would allow the systematic manipulation of all these factors in the same experiment. In Experiment 3 we attempted to identify the contribution of each of these factors using a larger 5 × 5 matrix of items (see also Kemps, 2001; Rossi-Arnaud et al., 2012; Cestari et al., 2013) that provided the flexibility for generating a sufficient number of sequences for a fully nested 2 (structure) × 2 (path-length) × 2 (crossings) factorial design.

### Methods

#### Participants

Twenty seven (20 female and 7 male, age range 19–27) undergraduate psychology students from the University of Leicester took part in Experiment 3 and received course credits for their participation. All participants reported normal or corrected to normal vision.

#### Materials, design, and procedure

**Figure 5:**
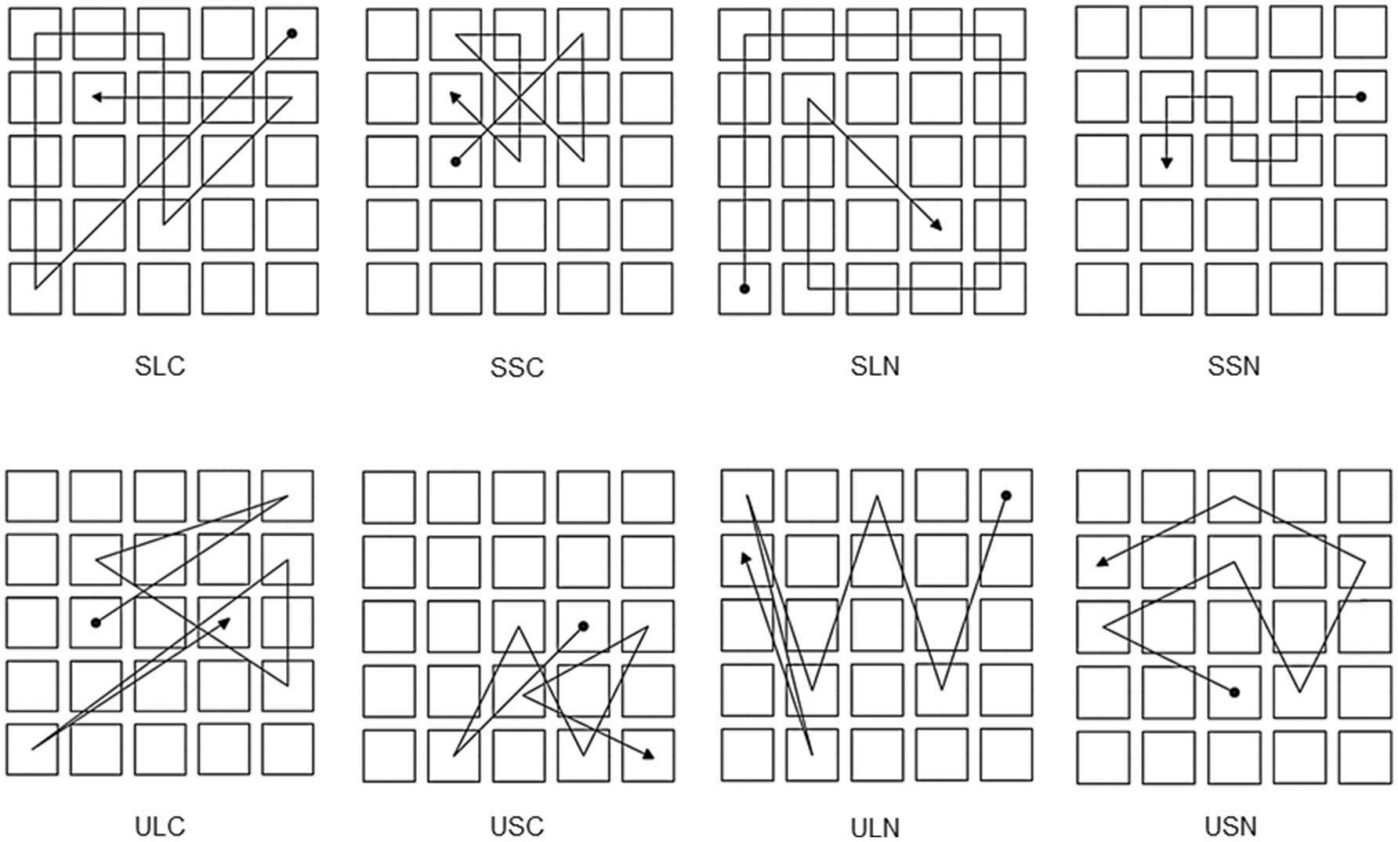
**Example of trials for the eight experimental conditions administered in Experiment 3: SLC, Structured Long with Crossings; SSC, Structured Short with Crossings; SLN, Structured Long No-crossings; SSN, Structured Short with No-crossings; ULC, Unstructured Long with Crossings; USC, Unstructured Short with Crossings; ULN, Unstructured Long No-crossings; and USN, Unstructured Short No-crossings**.

We used the same PC as in the previous two experiments. Software developed in-house allowed the presentation of 25 identical white squares arranged as a 5 × 5 matrix (see Figure 5). Each square had a side of 116 pixels (2.5 cm).

In each trial, participants were first presented with the 5 × 5 matrix of squares for 700 ms, before the sequence presentation started and the matrix of items remained present throughout the presentation and the recall phase of each trial. In this experiment the sequences contained 7 items. The timing of the item presentation was the same as in the first two experiments. A within-participant design was used. The independent variable was the type of sequence, defined according to its path characteristics (henceforth referred to as “sequence type”), with three factors: structure (structured/unstructured), path-length (long/short), and crossings (with/without crossings). Eight experimental conditions were obtained by nesting the two levels of each of the above factors: Structured Long with Crossings (SLC); Structured Short with Crossings (SSC); Structured Long No-crossings (SLN); Structured Short no Crossings (SSN); Unstructured Long with Crossings (ULC); Unstructured Short with Crossings (USC); Unstructured Long No crossings (ULN); and Unstructured Short No Crossings (USN). Examples of different types of sequences are presented in Figure 5.

An operational definition of the factors determining each type of sequence is provided below.

##### Structure

This was defined following Bor et al. (2003): “Structured sequences” had consecutive items within the same row, column or diagonal of the matrix; “Unstructured sequences” systematically violated these constraints (i.e., consecutive items were never within the same row, column, or diagonal).

##### Path-length

This was defined in terms of the total number of squares intersected by transitions between consecutive items in the sequence. Long sequences intersected a total of at least 10 items. Short sequences intersected a maximum of 6 squares.

##### Path-crossings

This variable referred to transitions in the sequence crossing over an imagined line of the previously completed sequence that the participants gaze or finger would travel through, as defined by Kemps (2001). Sequences without crossings did not contain any instance of the above and sequences with crossings contained at least three such crossings. The number of crossings in the sequence was chosen on the basis of previous research that reported sequences presented with three crossings having a significant negative effect on recall compared to sequences with no crossings. This effect did not continue linearly when more crossings were included (Parmentier et al., 2005).

The testing session comprised a total of 64 trials featuring 8 sequences for each of the 8 conditions, interspersed in random order.

### Results

**Figure 6:**
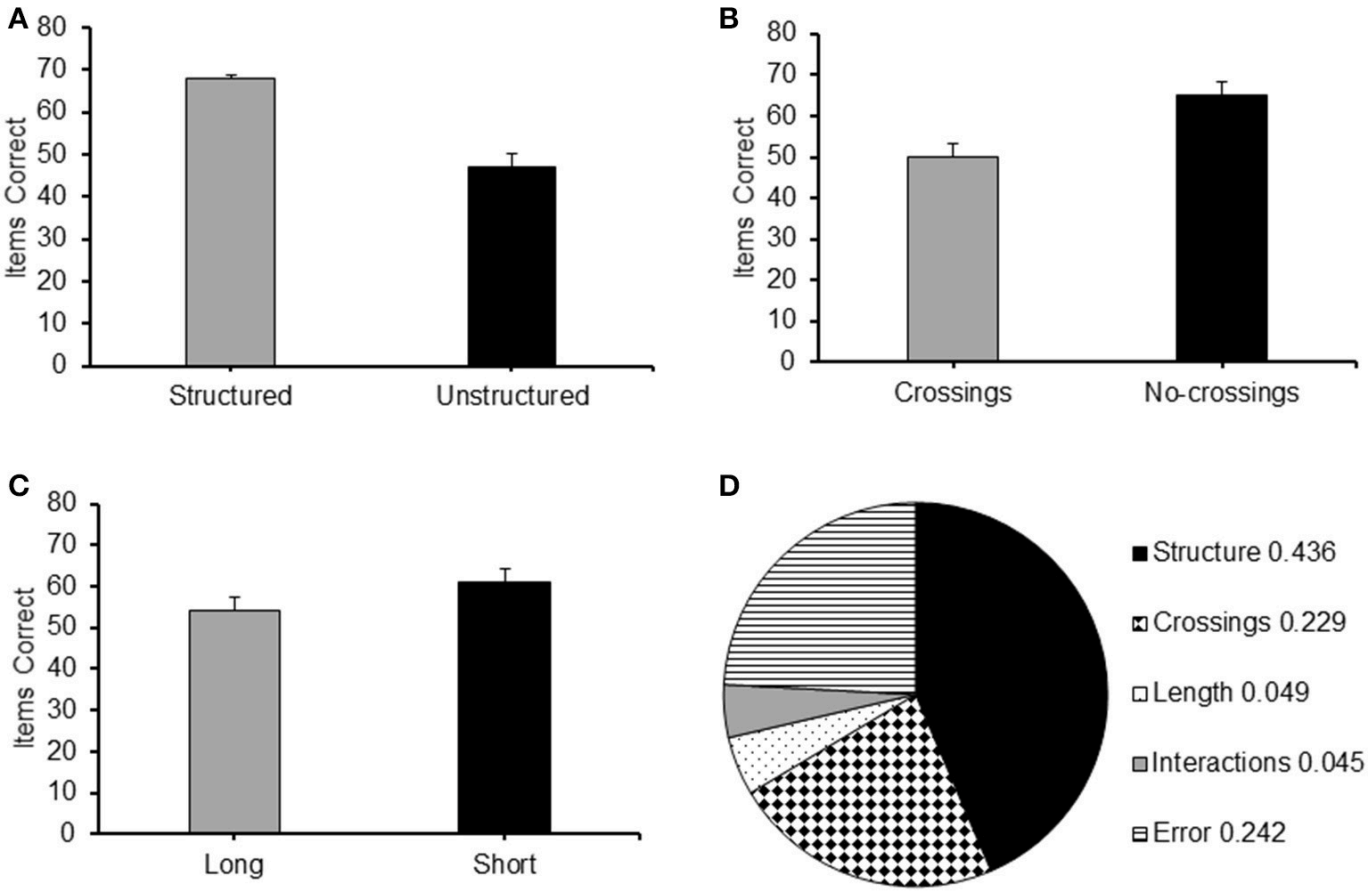
**Average frequency of items correct recalled for each level of the main factor of Experiment 3: (A)** Structure (Structured/Unstructured); **(B)** Crossings (Crossings/No-crossings) and **(C)** Path length (Long/Short); **(D)** Values of η^2^ indicating the proportion of total variance explained by Structure, Crossings and Path length, Interactions and error in the results of Experiment 3.

The main effects of the different conditions featured in Experiment 3 are presented in Figure 6. From the figure it can be observed that recall accuracy was better for sequences which were structured, or with a short path or without crossings.

A 2 (structure: structured/unstructured) × 2 (crossings: with/without crossings) × 2 (path-length: long/short) repeated measures ANOVA carried out on the frequency of items correctly recalled in the different conditions revealed significant main effects for all the three factors: structure [*F*_(1, 26)_ = 193.20, *p* < 0.001, $\eta_{\text{p}}^{2}$ = 0.881], with structured sequences recalled with a higher level of accuracy than non-structured sequences (see Figure 6A); crossings [*F*_(1, 26)_ = 132.60, *p* < 0.0005, $\eta_{\text{p}}^{2}\;=$ 0.836] with sequences without crossings recalled at a higher level of accuracy than sequences with crossing (see Figure 6B); and path-length [*F*_(1, 26)_ = 54.69, *p* < 0.001, $\eta_{\text{p}}^{2}=0.678$], with short sequences reported at a higher level of accuracy than long sequences (see Figure 6C).

The only significant second order interaction was between the factors structure and crossings [*F*_(1, 26)_ = 18.807, *p* < 001, $\eta_{\text{p}}^{2}\;=$ 0.42]. However, *t*-tests comparing accuracy for sequences with and without crossings, with the two levels of the factor path-length combined, all revealed highly significant differences [Structured-No crossings vs. Structured-with-Crossings, *t*_(26)_ = 9.503, *p* < 001; Unstructured-No crossings vs. Unstructured-with-Crossings, *t*_(26)_ = 6.25, *p* < 0.001]. Therefore, this interaction cannot be explained by the lack of an effect of crossings at some level of the factor structure. Instead, the interaction is likely to be due to a slight variation of the effects of crossings at the two levels of sequence structure. In fact, whereas the difference in the accuracy of recall in the sequence with and without crossings was of 21.15 in the structured condition, it was of only 9.37 in the unstructured condition. The accuracy values for the sequences with crossings and without crossing for structured and unstructured sequences are reported in Table 1. A significant third order interaction also emerged [*F*_(1, 26)_ = 4.745, *p* < 0.05, $\eta_{\text{p}}^{2}=$ 0.154]. Planned comparisons (*t*-tests with Bonferroni correction, alpha = 0.0042) of the effects of each of the factors with each level of the other factors kept constant, revealed all significant effects [4.21 < *t*_(26)_ <12.03, *p* < 0.05] with the exception of the comparison between sequences with and without crossing in the unstructured short sequences [*t*_(26)_ = 2.59, *p* = n.s.] and the difference between long and short path-length in the unstructured sequences with no crossings [*t*_(26)_ = 1.05, *p* = n.s.].

**Table 1:** **Experiment 3: participants mean frequency of correct items and Standard Deviation for on each type of sequence**.

	**Structured**		**Unstructured**	
	**Crossings**	**No crossings**	**Crossings**	**No crossings**
Long	55.56 (*SD* 21.12)	74.11 (*SD* 18.75)	37.67 (*SD* 12.72)	51.03 (*SD* 19.37)
Short	61.63 (*SD* 18.60)	83.37 (*SD* 18.97)	47.19 (*SD* 18.47)	52.56 (*SD* 16.78)

#### Eta squared

In order to illustrate the strength of the experimental effect of each of the three factors, in Figure 6D, we report the value of the η^2^ which indicates the proportion of variance that is explained by each of them (see Howell, 2002). As can be seen from the figure, path-crossings explained a larger portion of variance than path-length. Importantly, the largest portion of variance is explained by residual effects of structure that cannot be explained by either path-crossings or path-length.

#### Serial position analysis

**Figure 7:**
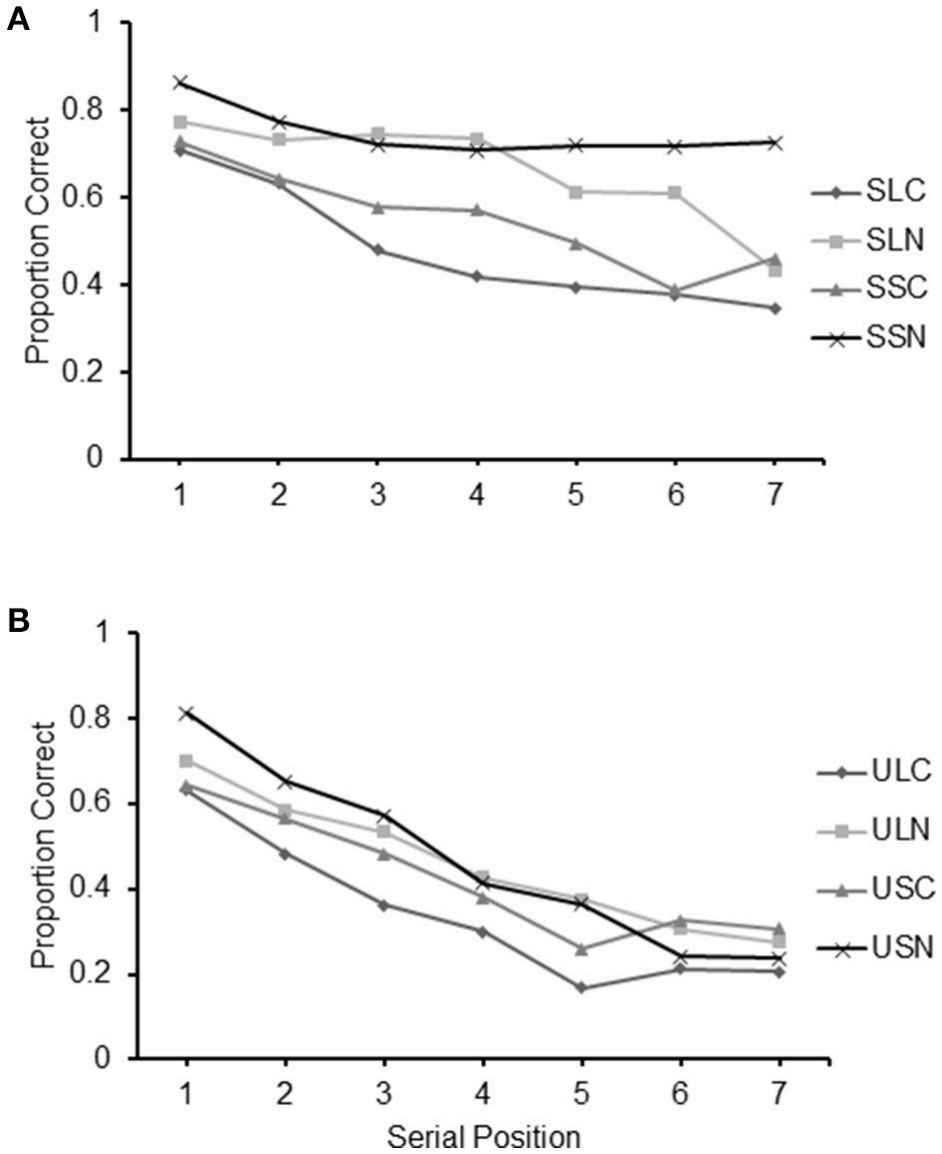
**Proportion of correct items recalled at each serial position for the eight conditions of Experiment 3: (A)** SLC, Structured Long with Crossings; SSC, Structured Short with Crossings; SSN, Structured Long No-crossings; **(B)** ULC, Unstructured Long with Crossings; USC, Unstructured Short with Crossings; ULN, Unstructured Long No-crossings; and USN, Unstructured Short No-crossings.

Serial position curves for all conditions are reported in Figure 7. For clarity the curves are presented separately for conditions featuring structured sequences (Figure 7A) and those featuring unstructured sequences (Figure 7B). An ocular inspection of the figure indicates the presence of serial position effects in all conditions. Albeit present in all curves, a decreasing level of recall in relation to the serial position of the items is particularly evident in conditions featuring unstructured sequences.

A 7 (serial position: 1/2/3/4/5/6/7) × 2 (structure: structured/unstructured) × 2 (path-length: long/short) × 2 (crossings: with/without crossings) repeated measures ANOVA confirmed the presence of a main effect of serial position [*F*_(6, 156)_ = 86.81, *p* < 0.001, $\eta_{\text{p}}^{2}=$ 0.770] in addition to the effects of structure [*F*_(1, 26)_ = 193.33, *p* < 0.001, $\eta_{\text{p}}^{2}=$ 0.881], path-length [*F*_(1, 26)_ = 54.87, *p* < 0.001, $\eta_{\text{p}}^{2}=$ 0.678], and crossings [*F*_(1, 26)_ = 132.99, *p* < 0.001, $\eta_{\text{p}}^{2}=$ 0.836]. The interactions Structure by Crossings [*F*_(1, 26)_ = 18.87, *p* < 0.001, $\eta_{\text{p}}^{2}$ = 0.421], Structure by Path-length by Crossings [*F*_(1, 26)_ = 4.83, *p* < 0.05, $\eta_{\text{p}}^{2}$ = 0.157], Structure by Serial position [*F*_(6, 156)_ = 19.85, *p* < 0.05, $\eta_{\text{p}}^{2}$ = 0.443], Path-length by Serial position [*F*_(6, 156)_ = 3.37, *p* < 0.05, $\eta_{\text{p}}^{2}$ = 0.115], Structure by Path-length by Serial position [*F*_(6, 156)_ = 6.42, *p* < 0.001, $\eta_{\text{p}}^{2}$ = 0.198], Crossings by Serial position [*F*_(6, 156)_ = 6.34, *p* < 0.001, $\eta_{\text{p}}^{2}$ = 0.196], Structure by Crossings by Serial position [*F*_(6, 156)_, *p* < 0.001, $\eta_{\text{p}}^{2}$ = 0.308], Path-length by Crossings by Serial position [*F*_(6, 156)_ = 5.81, *p* < 0.001, $\eta_{\text{p}}^{2}$ = 0.183], Structure by Path-length by Crossings by Serial position [*F*_(6, 156)_ = 6.18, *p* < 0.001, $\eta_{\text{p}}^{2}\;$ = 0.192] were all significant.

Although it is difficult to pinpoint the exact nature of these complex interactions, they are likely to be due to various small variations occurring at different points of the serial position curve of the different conditions that otherwise showed a relatively similar pattern. In fact, trend analyses carried out individually for the different conditions revealed that they all had a significant linear component [9.95 < *F*_(6, 156)_ <165.39, all ps <0.001, 2.77 < $\eta_{\text{p}}^{2}$ <0.864] that could be indicative of a primacy effect as performance deteriorated in line with the serial position of the items in the sequence. The quadratic component of the trend was highly significant in conditions SLC, SLN, SSN, ULC [16.28 < *F*_(1, 26)_ <45.87, 0.374 < $\eta_{\text{p}}^{2}$ <0.481] and USC and significant in condition USN [*F*_(1, 26)_ = 6.79, *p* < 0.05, $\eta_{\text{p}}^{2}$ = 0.205]. It was not significant in conditions SSC and ULN.

The graphs suggest the lack of a recency effect, apart from increases in recall between items 6 and 7 in condition SSC, *t*_(26)_ = 2.92, *p* < 0.01 and between items 5 and 6 in conditions USN, *t*_(26)_ = 4.43, *p* < 0.001 and USC, *t*_(26)_ = 2.64, *p* < 0.05, that may be indicative of such an effect.

### Discussion

In Experiment 3 we carried out a systematic manipulation of sequence structure as previously defined in the literature (Bor et al., 2003) and two path characteristics that have been shown to affect SSR (Kemps, 2001; Orsini et al., 2001, 2004; Parmentier et al., 2005; Parmentier and Andrés, 2006; Fagot and De Lillo, 2011). The effects of structure and path characteristics can be confounded (see Parmentier et al., 2005, 2006; De Lillo and Lesk, 2010). Thus, the aim of Experiment 3 was to evaluate whether or not the effects of path characteristics can explain in their entirety purported effects of structure. The results indicate that they cannot. The experiment confirmed the presence of the effects of path characteristics reported in the literature, namely, path-length and path-crossings (Parmentier et al., 2005, 2006). However, the results suggest that beneficial effects related to structure played a role on top of effects related to path characteristics. Moreover, the effects of structure emerged as the one explaining most of the variance in the data, followed by effects of path-crossings and finally by path-length effects.

As outlined above, the literature on organizational factors in SSR, often refers to the possible role of perceptual grouping factors in relation to the beneficial effects of structure (Kemps, 1999; Bor et al., 2003; Rossi-Arnaud et al., 2005; Ridgeway, 2006; Bor, 2012; Hurlstone et al., 2014). Perceptual grouping effects occurring at the time of observing the to-be-reproduced sequence could possibly take place in SSR tasks affording an aerial view of items presented in a rapid sequence. In situations where participants are required to slowly navigate through the items to apprehend the sequence to be reproduced the occurrence of perceptual grouping is much less likely. In Experiment 4 we aimed to assess if effects of structure emerge in navigational tasks too.

## Experiment 4

In Experiment 4, we used a clustered configuration similar to the one used in Experiments 1 and 2 and a 3 × 3 matrix of locations. This allowed a direct comparison of the effects of structure and path-length with the two configurations and different operational definitions of structure. Importantly, however, SSR was assessed as part of an immersive virtual reality navigational task that required changes of viewpoint and slow movements by the participant to apprehend consecutive items in the sequence (see Supplementary Video). Visual perceptual grouping normally occurs between elements that are presented as part of the same visual display viewed from a fixed viewpoint. Albeit recent evidence suggest a different timescale for the processing of different stages of grouping, visual grouping processes seem to be completed within a time range spanning a few hundred milliseconds to a second (Kurylo, 1997; Han et al., 1999, 2002; Brick Larkin and Kurylo, 2013). Thus, we reasoned, if effects of structure in SSR emerge when the to-be-recalled sequence is presented as a part of a large scale navigation task, lasting over a minute and requiring continuous changes of viewpoint, then it is unlikely that they are caused by perceptual grouping processes.

### Methods

#### Participants

Twenty one participants (10 females and 11 males, age range 18–42) took part in the experiment. They were psychology undergraduates, who received course credits for participation, or members of a participant panel, comprising mainly of post-graduate students and staff, who were paid a small fee for taking part.

#### Materials, design, and procedure

**Figure 8:**
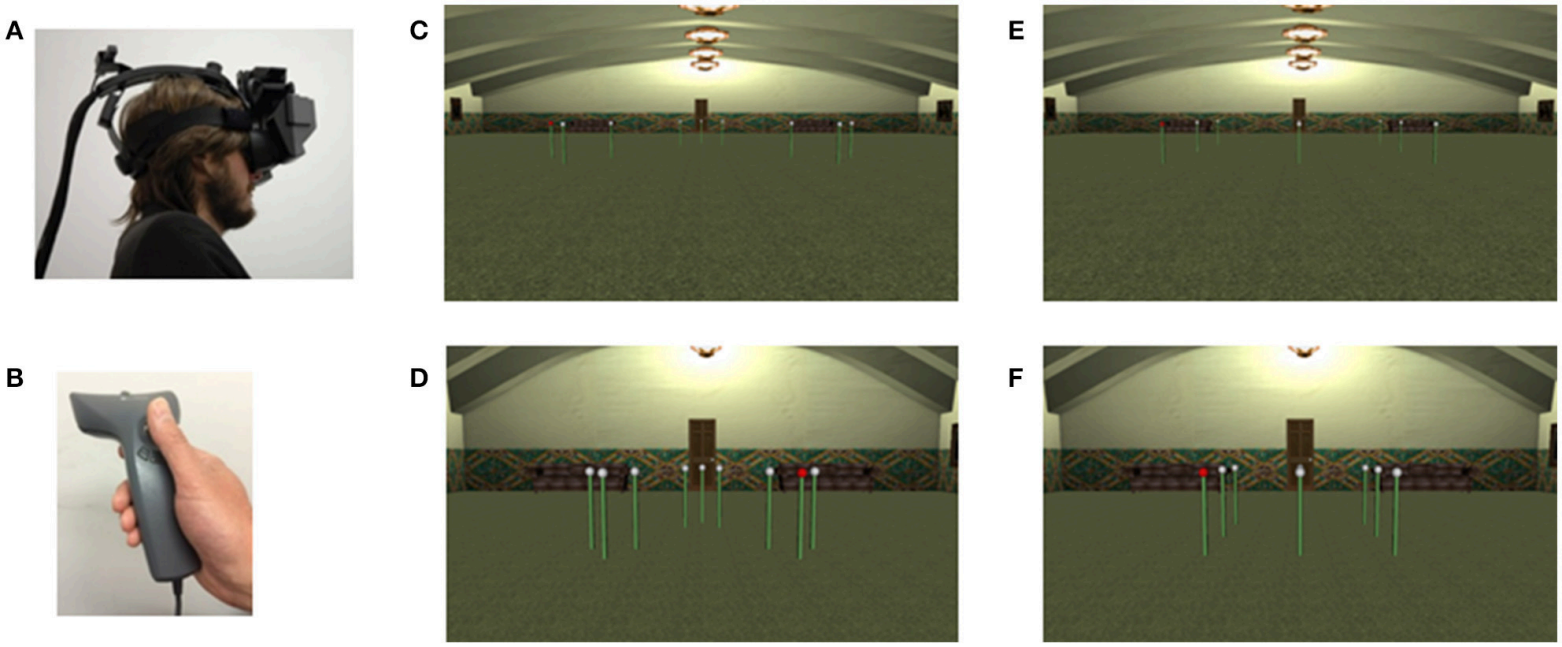
**Virtual Reality (VR) set-up and displays used in Experiment 4: (A)** headset used to display the task; **(B)** wand used for navigating and selecting poles in the VR environment; **(C)** large clustered configuration; **(D)** small clustered configuration; **(E)** large matrix configuration; **(F)** small matrix configuration.

The experiment took place in a Virtual Reality laboratory equipped with an NVIS nVisor stereoscopic head mounted display (see Figure 8A). An Inter-Sense position tracker determined the viewpoint depending on the head and the body movement of the participants who operated a hand held wand to navigate and produce responses. Traveling in the virtual environment was controlled by moving with the thumb a small joystick located on the wand (see Figure 8B). Traveling speed was set to 1 meter per second. The software was developed in house using Vizard 3.0. (WorldViz) and allowed the presentation of a virtual environment consisting of a set of 9 poles surmounted by a white sphere within a large virtual hall with richly textured surfaces and several landmarks (see Figures 8C–F).

**Figure 9:**
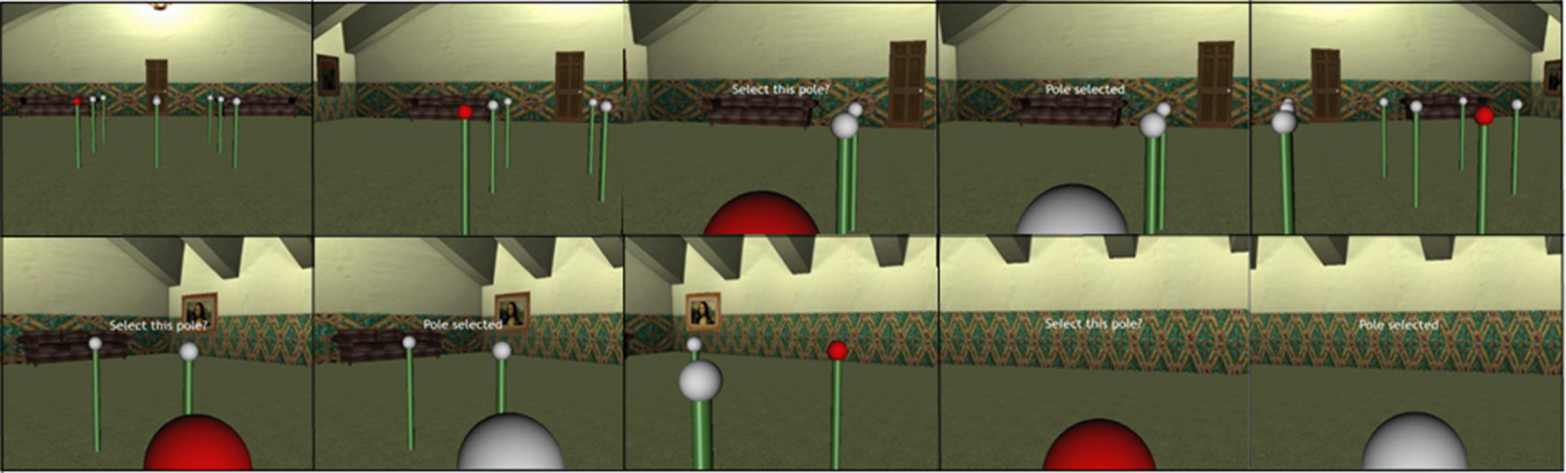
**Examples of viewpoints experienced during the presentation phase of a trial of Experiment 4**. The figure shows that approaching the required pole, selecting it, it and then turning within the virtual reality environment to determine the location of the next pole in the to-be-recalled sequence required dramatic changes of view point. This made it impossible for visual perceptual grouping to contribute to effects of structure in this implementation of a serial recall task.

The general serial recall procedure was implemented as described below. Each trial featured a presentation phase and a recall phase. In the presentation phase, one of the white spheres surmounting the poles turned red until the participant traveled through the environment, approached and selected it by operating the wand. As soon as the pole was selected it turned white again and a second pole within the array turned red, and so on, until all the 9 poles had been selected by the participant. Thus, the selection of consecutive items during the presentation of the sequence required the participants to search and navigate toward different points of the environment. Since traveling time was set at 1 meter per second and it could take time for participants to rotate their head and identify the next item to reach, the presentation of the sequence was a lengthy process sometimes lasting one or more minutes to complete. Following the completion of the presentation phase, the recall phase immediately ensued. All poles turned white. The starting position was reinstated and the participant had to recall the sequence by navigating through the virtual environment and selecting the poles in the same order as in the presentation phase. The head movement of the participant was tracked by the inter-sense system and used to update the view-point producing a vivid immersive experience, and importantly determining that the viewpoint changed continuously (see Figure 9, and Supplementary Video, for a an example of viewpoint experienced during the presentation of the sequence), depending on the position of the head and the body of the participant throughout the presentation of the sequence.

The design comprised a within subjects manipulation of the configuration of poles (clustered or matrix), the structure of the sequence (structured or unstructured), and the distance between the poles path-length (long and short) of the sequence to be recalled. The configurations and display sizes are shown in Figures 8C–F.

Configuration and path-length were manipulated by changing the spatial arrangement and the distance of the poles, respectively. Depending on the configuration condition the poles were arranged as a 3 × 3 square matrix or a clustered configuration. The path-length was manipulated by making the inter-pole distance in the long path condition 3 times longer than the distance in the short path condition. In particular, the minimum possible distance between the poles was 2.1 m in the long path condition and 0.7 m in the short path condition. The starting point from the center of the configuration of poles was 14.7 and 4.9 m in the short-path condition. For the clustered configuration, the structure of the sequence was manipulated as in Experiment 1. For the matrix configuration, it was manipulated as in Experiment 3. Participants received alternating trials of the short-path and the long path condition, with the starting position randomized across participants. Apart from this constraint, the conditions were randomized across trials. Each participant received two trials per condition for a total of 16 trials. Because of the weight of the headset, to ensure comfort, participants were given a short break every two trials, when required, and a 10 min break after eight trials.

### Results

**Figure 10:**
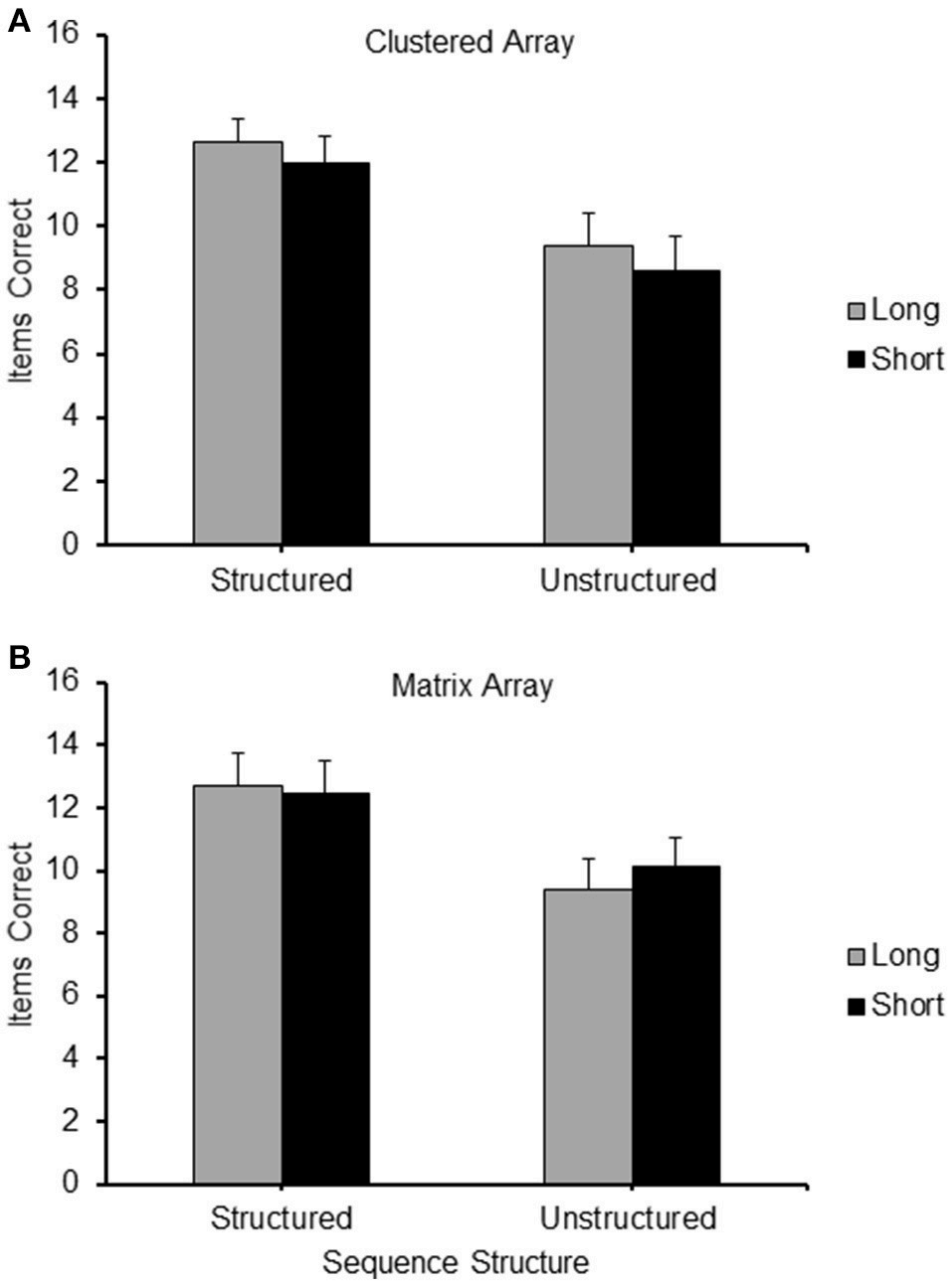
**Frequency of items correct recalled for the different conditions (Long Structured; Short Structured; Long Unstructured and Short Unstructured) of Experiment 4, for the Clusters (A)** and Matrix **(B)** layout of locations.

The mean frequency of items correctly recalled in the different conditions of Experiment 4 is depicted in Figure 10A (Clustered configuration) and Figure 10B (Matrix configuration).

A 2 (Configuration: clusters/matrix) × 2 (Path-length: long/short) × 2 (Structure: structured/unstructured) repeated measure ANOVA carried out on the frequency of items correctly recalled in the different conditions revealed a highly significant main effect for structure [*F*_(1, 20)_ = 49.86, *p* < 0.001] and a significant main effect of configuration [*F*_(1, 20)_ = 6.94, *p* < 0.05], where structured sequences were recalled significantly more accurately than unstructured sequences, and sequences in the matrix configuration were recalled slightly more accurately than the clustered configuration. By contrast, there was no significant difference in recall performance between long and short path-length [*F*_(1, 20)_ = 0.021, *p* = n.s.]. None of the interactions proved significant.

Similarly to the analysis of the results of Experiment 1, we carried out the critical comparison between the Long Structured and the Short Unstructured conditions in using paired sample *t*-tests on the mean frequency of correct items observed in these two conditions. The means for these conditions are reported as part of Figure 10. The observed level of accuracy was higher in the long structured condition compared to the short unstructured condition in both the clustered [*t*_(20)_ = 2.44, *p* < 0.05] and the matrix configuration [*t*_(20)_ = 4.04, *p* = 0.001].

Because each condition only featured two trials in Experiment 4, the data set generated for this experiment was not suitable for serial position analyses.

### Discussion

The results of Experiment 4 confirm the main findings of Experiment 1. Moreover, they show that participants can benefit from effects of structure even when they do not have a bird's eye view of the configuration of items. Importantly, the view point of the participants changed continuously during the task, as a function of their position within the virtual environment and mostly afforded seeing only a portion of the configuration of poles. Furthermore, each presentation phase lasted at least 1 min. Thus, the task characteristics, both in terms of viewpoint and timescale, make it unlikely that visual perceptual grouping mechanisms underpin the benefits of structure in this task. Additionally, the results of Experiment 4 confirmed that path-length has a marginal role in serial recall, even in conditions that should exacerbate its role, as in navigation. This pattern of results seems to be very robust and confirms the results of other experiments carried out with a similar procedure but with a different and smaller sample of participants (De Lillo et al., 2014).

Experiment 4 did not address specifically the role of path-crossings in situations where participants do not have a bird's eye view of the configuration of test items. This was because Experiment 4 already featured several variables and because it is not possible to systematically vary path-crossings and distance at the same time in the clustered configuration. Therefore, the effects of this variable in relation to traveling distance was addressed in Experiment 5 by focusing exclusively on the matrix configuration and using in this VR navigational environment a design similar to that used in Experiment 3.

## Experiment 5

### Methods

#### Participants

Twenty participants (10 female and 10 male, with a mean age of 19.90, *SD* = 2.56) undergraduate psychology students from the University of Leicester took part in Experiment 5 and received course credits for their participation. All participants reported normal or corrected to normal vision.

#### Materials, design, and procedure

The apparatus used was the same as described for Experiment 4. The VR environment was similar to that used for Experiment 4 and the administration of the trials followed the same procedure. However, the configuration of poles in the VR environment and the design of the experiment were modeled on those of Experiment 3. Thus, Experiment 5 featured a 5 × 5 matrix of items and the same eight experimental conditions used for Experiment 3: Structured Long with Crossings (SLC); Structured Short with Crossings (SSC); Structured Long No-crossings (SLN); Structured Short No-crossings (SSN); Unstructured Long with Crossings (ULC); Unstructured Short with Crossings (USC); Unstructured Long No-crossings (ULN); and Unstructured Short No-crossings (USN). Examples of different types of sequences are presented in Figure 5. Because of the length of the testing session and the weight of the head mounted display, only two sequences for each of the conditions was presented to the participants, as in Experiment 4. The sequences were pseudo-randomly selected from the pool of sequences for each participant and each presentation with the constraint that the first eight trials had to feature one of each of the conditions. After the presentation of the first eight trials the participants were given the opportunity to take a short break before being presented with the second trial of all the conditions.

### Results

**Figure 11:**
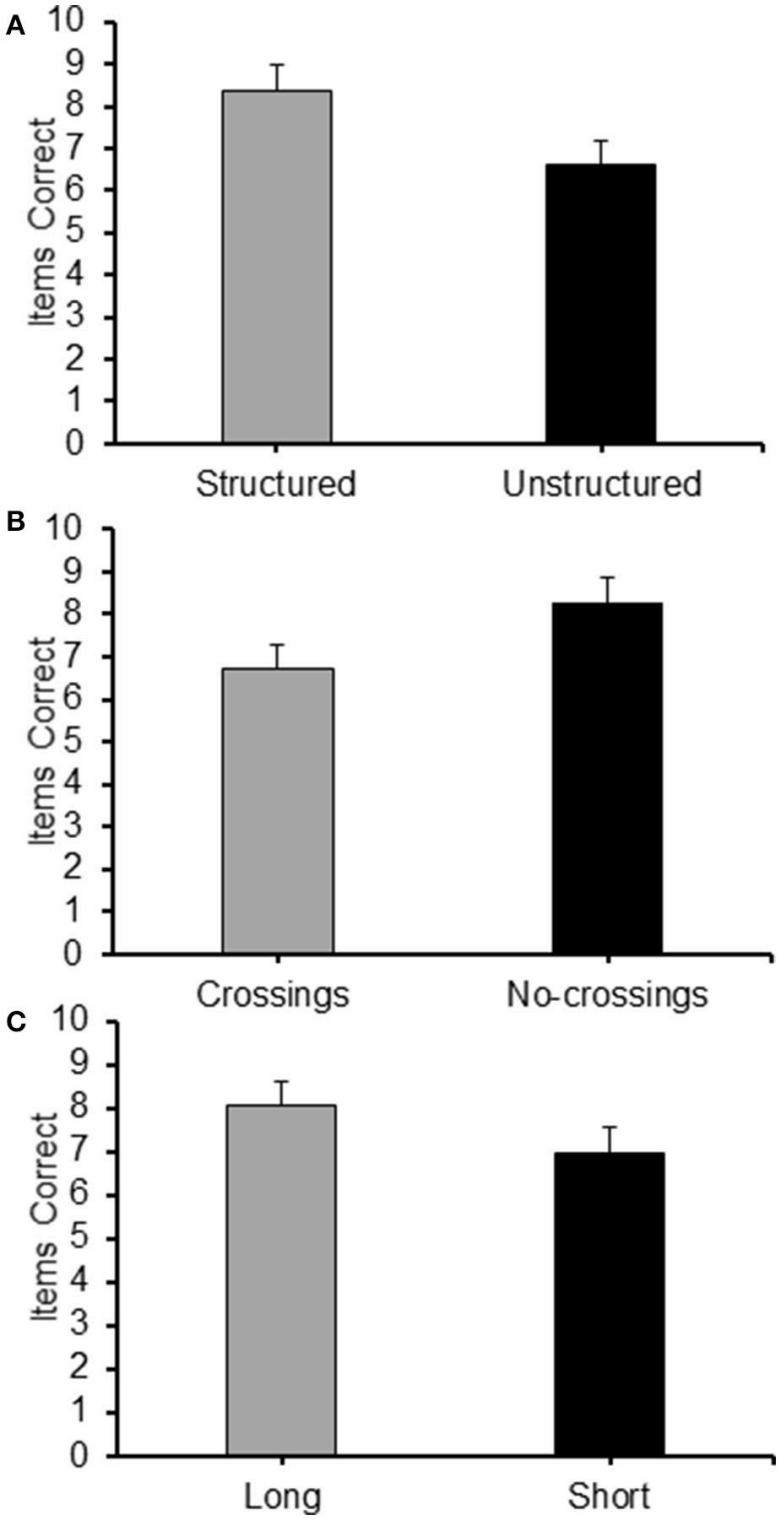
**Average frequency of items correct recalled for each level of the main factor of Experiment 5: (A)** Structure (Structured/Unstructured); **(B)** Crossings (Crossings/No-crossings); and **(C)** Path length (Long/Short).

As for Experiment 3, a 2 (Structure: structured/unstructured) × 2 (Crossings: with/without crossings) × 2 (Path-length: long/short) repeated measures ANOVA carried out on the frequency of items correctly recalled in the different condition revealed significant main effects for all the three factors: structure [*F*_(1, 19)_ = 11.66, *p* < 0.01, $\eta_{\text{p}}^{2}$ = 0.380], with structured sequences recalled with a higher level of accuracy than non-structured sequences (see Figure 11A); crossings [*F*_(1, 19)_ = 14.73, *p* = 0.001, $\eta_{\text{p}}^{2}=$ 0.437] with sequences without crossings recalled at a higher level of accuracy than sequences with crossings (see Figure 11B); and path-length [*F*_(1, 19)_ = 7.69, *p* < 0.05, $\eta_{\text{p}}^{2}$ = 0.288], with longer sequences reported at a higher level of accuracy than shorter sequences (see Figure 11C). None of the interactions proved significant.

#### Eta squared

As for Experiment 3, η^2^ were calculated in order to evaluate the proportion of variance explained by the different factors. Albeit the values of η^2^ for the main effects were smaller than those observed in Experiment 3, they followed the same pattern. Structure accounted for most of the total variance (η^2^ = 0.084), followed by crossings (η^2^ = 0.066), and finally path-length (η^2^ = 0.034). The values of η^2^ obtained for the error and the interactions were η^2^ = 0.760 (error) and η^2^ = 0.055 (interactions), respectively.

As only 2 trials were collected for each condition, it was not feasible to carry out a serial position analysis for this experiment.

### Discussion

The results of Experiment 5 confirmed those obtained in Experiment 3. They indicate that the effects of structure and path characteristics are very robust and emerge in conditions where the viewpoint of the participants changed continuously during the presentation of the sequence and during recall. For these effects to emerge it is not necessary to have a bird's eye view of the display. This finding is particularly important in relation to the effects of structure. It confirmed that the effect of structure cannot be entirely explained by path characteristics alone and also that it can emerge when the operation of perceptual grouping principles is unlikely.

## General discussion

We have reported five experiments exploring the contribution of different factors to immediate SSR. We assessed effects of path-length, path crossing and visual perceptual organization and determined the presence of residual effects of stimulus structure which are beneficial to recall but cannot be attributed to these factors.^1^

Path-length and path-crossings have been previously shown to affect spatial span as measured by the standard Corsi test (Orsini et al., 2001, 2004) and the related dot task (Parmentier et al., 2005; Parmentier and Andrés, 2006; Guerard and Tremblay, 2012). Because these tests of immediate spatial recall use irregular arrangements of items it is difficult to determine with them which structural affordances of the display participants may detect and use to facilitate the recall of given sequences. Hence, the need to use spatial layouts of items specifically designed to allow, or prevent, forms of spatiotemporal organization which are known a priori to afford memory coding of a particular kind.

For these reasons, our first two experiments used a Corsi-type task featuring a configuration of identical icons arranged as spatial clusters defined by the relative proximity of the items (De Lillo, 2004; Parmentier et al., 2006; De Lillo and Lesk, 2010). Within this type of clustered configuration, sequences that are segregated by clusters afford hierarchical memory coding (De Lillo, 2004; De Lillo and Lesk, 2010). However, effects of spatial clustering there can be confounded by path-length. In fact, it has been suggested that path-length alone could explain improved recall in such displays (Parmentier et al., 2006).

The results of Experiment 1 showed a dissociation between path-length and structure defined as segregation by spatial clusters. Structure proved an important determinant of spatial recall performance. By manipulating path-length in large and small arrays of spatial items (see also Smyth and Scholey, 1994) we showed that there are effects of segregation by clusters which are independent from, and stronger than, path-length. In fact, in Experiment 1 structured sequences were recalled at a higher level of accuracy than non-structured sequences irrespectively of path-length.

Although this finding is consistent with the notion that the segregation of sequences by spatial clusters affords hierarchical memory representations of the sequences that facilitates recall (De Lillo, 2004), in Experiment 1 it was not possible to test this possibility directly. Moreover, because most sequences segregated by clusters have fewer crossings than unstructured sequences, it was not possible to determine in Experiment 1 whether or not there are effects of segregation by clusters which can be attributed to hierarchical coding, in addition to possible beneficial effects deriving from the presence of fewer crossings in these sequences.

In Experiment 2 we controlled for the effects of crossings and tested the hierarchical coding hypothesis of the benefits of clustering in SSR, by exclusively using sequences segregated by clusters and manipulating the temporal phrasing of sequence presentation. Critically, Experiment 2 provided independent evidence that recall in sequences segregated by spatial clusters is supported by hierarchical coding. A lower level of recall was observed in conditions featuring the insertion of pauses within cluster boundaries and which were not compatible with a retrieval process that exploited such organization. In this respect, our results conform to those of studies that manipulated the presentation of pauses within and between hypothesized chunks to infer the presence of hierarchical coding in the spatial (Bor et al., 2003) and other domains (Farrell and Lelievre, 2012).

Thus, taken together the results of the first two experiments provide synergistic support for the notion that the beneficial effects of segregating sequences by spatial clusters derives from hierarchical coding in spatial working memory. The results are also consistent with evidence of hierarchical organization in SSR obtained in studies where the size and number of spatial clusters were manipulated in conditions otherwise similar to those of the present study (De Lillo and Lesk, 2010). There emerged that RT at cluster boundaries is proportional to the number of items in the cluster, reflecting the time taken to access the order of report of the items within that cluster (subordinate level of the hierarchy). By contrast, a component of the initial RT, which was deemed to be an expression of the time taken to retrieve the order of report of the clusters into which the sequence was segregated, was proportional to the number of clusters (superordinate level of the hierarchy). Albeit consistent with those of previous studies featuring clustered Corsi arrays (De Lillo, 2004; De Lillo and Lesk, 2010), the results of Experiment 1 and 2 provide crucial information concerning whether or not path-length could account on its own for the improved recall observed in sequences segregated by spatial clusters. The results show that this is not the case.

In clustered arrays such as those used in the first two experiments (as well as in irregular arrays, see Ridgeway, 2006) it is difficult to systematically manipulate the presence of path-crossings and path-length independently. Therefore, in our Experiment 3 we used a matrix of locations where it was possible to do so. We constructed sequences with nested characteristics so that a fully factorial design could be used to assess the portion of variance in the data explained by each of these factors. The results confirmed the presence of effects of all these factors with residual positive effects of structure. Structure was defined as in other studies that have used a square matrix of locations to administer a Corsi-type tasks (Bor et al., 2003). The presence vs. absence of structure in the sequences produced the largest portion of variance in recall. This suggests that principles other than path-length and crossings are most important in determining ease of recall in serial spatial memory.

Compared to spatially clustered arrays, matrices of locations make it more difficult to infer the reason for the benefits of structure defined as having consecutive serial spatial items within the same column, row or diagonal in a square matrix of locations. Bor et al. (2003) proposed that the benefits are derived from chunking and are based on gestalt principles. Similar claims have been made in other studies where gestalt principles such as continuity and symmetry were explicitly applied to the sequences (Kemps, 2001; Rossi-Arnaud et al., 2005). Reference to the formation of gestalten that facilitate SSR raises the question of whether perceptual grouping principles need to operate during the presentation of the sequences for this to occur.

The position of several authors converge on the notion that perceptual processes may account for the formation of chunks at encoding (Kemps, 2001; Bor et al., 2003; Parmentier et al., 2005; Avons, 2007; Bor, 2012). When reviewing these studies Bor (2012) queried whether the process of organizing the to-be-remembered material occurs “on-the-fly” that is, “spotting the pattern as a powerful new rule to apply on each trial” (Bor, 2012, p. 179). On the basis of the fact that an activation of the fusiform gyrus occurs in addition to the activation of the DLPFC in trials featuring structured sequences, Bor et al. (2003) suggested that chunking in this domain may be related to the object perception functions of this cortical region. A study based on self-reports of participants presented with variations of the Corsi test has attempted to dissociate what the author refers to as perceptual grouping and strategic grouping in the assessment of spatial span (Ridgeway, 2006). The study concluded that both perceptual and strategic grouping occur and give rise to enhanced memory performance in this task. This conclusion was based on the fact that some participants reported the use of strategies consisting of dividing the sequence to be reported in subgroups even in sequences where a clear-cut separation between some of its sub-sequences was not present. This is a potentially important distinction. Nevertheless, so far the relative contribution of each of these two types of grouping to serial recall accuracy has not been clarified.

On the basis of these considerations we deemed it important to determine whether or not perceptual grouping is essential for the benefits of structuring in spatial working memory to occur. We addressed this issue in our last two experiments by assessing the effects of structure and path characteristics in conditions that made perceptual grouping unlikely to occur.

The immersive virtual reality serial recall task used in Experiments 4 and 5 was designed so that participants would navigate within the configuration of items forming the display. As a navigational version of the Corsi test, the VR task developed here is similar to walking Corsi tasks where participants observe a model walking along a route connecting locations in a real-life environment that they are required subsequently to reproduce (Piccardi et al., 2008, 2013; Nemmi et al., 2013). The walking Corsi has proved a very useful diagnostic tool for the detection of specific topographical memory deficits and sex differences in spatial memory (Piccardi et al., 2013) but has not been used to assess the role of organizational factors in WM. Our study differs from those featuring the walking Corsi as we used structured arrays of locations where different types of sequences were systematically manipulated rather than irregular arrays and sequences resembling those used in the traditional Corsi test.

Moreover, the virtual reality task used here differs in important aspects to the walking Corsi. During the observation of the sequences in the walking Corsi the observer stands at a distance from the array of locations traveled by the model. Thus, the entire sequence is seen from a single vantage point outside the configuration that the participant will be required to explore. Finally, the set of locations featured in the walking Corsi test are marked on the floor, making the test very similar to the standard Corsi and very useful for the comparison of spatial span in reaching and navigational space (Nemmi et al., 2013). In contrast with the walking Corsi, in our tasks during the presentation of the sequences participants were required to approach each item in the sequence and select it. Only then, a cue concerning the location of the next item was displayed in the navigational space. Thus, the entire sequence could never be observed from a single vantage point. The use of a head mounted display with head trackers ensured that the viewpoint of the participants changed at every movement the head performed to scan the array to search for the item to approach. This, together with the virtual movement throughout the array of items and the timing of the selection of each item, made it unlikely that participants could rely on visual grouping to form chunks. Yet, in Experiment 4 we still observed facilitating effects of structure in both the clustered and the matrix arrangement of items and in sequences characterized by either a short or long path. Moreover, even in a situation which made longer movements particularly costly in terms of time and distance traveled, path-length could not explain on its own the benefit of structure. In fact, as in Experiment 1, participants recalled more accurately structured long path sequences than unstructured short path sequences.

In Experiment 5 we used a design similar to that of Experiment 3 and simultaneously manipulated structure, path-length, and crossings in a VR environment. It emerged again that the effect of structure can be dissociated from the effect of the other two factors and explains a larger portion of variance than crossings and path-length. Path-length had a small effect and counterintuitively sequences with a longer path-length were recalled slightly more accurately than sequences with shorter paths. This indicates once again that beneficial effects of structure cannot always be reduced to the shortening of path-length often associated with structured sequences. A possible reason for the small advantage for sequences with longer path-length in this experiment is that a longer distance, on average, between consecutive items makes it easier for the participants to encode items as pertaining to distinctive sub-regions of space in the environment.

Interestingly, it has been proposed that path-length effects in spatial recall tasks presented on computer monitors that afford an aerial view of the display could be related to perceptual grouping processes. Longer path-length would hinder grouping principles that otherwise strengthen the coding of the transition between successive items of sequences and result in less accurate recall (Guerard and Tremblay, 2012). The lack of a positive effect of shorter path-length in our navigational serial recall tasks used to prevent grouping could be consistent with this theoretical interpretation of path-length effects.

Most importantly, taken together, the results of Experiments 4 and 5 indicate that visual perceptual grouping processes do not need to occur for the benefit of structure in serial recall to emerge. Thus, the encoding of structure is likely to have occurred at a post perceptual stage of processing. Our results are neutral to the issues of whether these effects of structure occur during encoding, rehearsal or recall. Yet there is evidence for the fact that they are likely to occur at encoding. For example, in the study by Bor et al. (2003) the selective of the dorsolateral prefrontal cortex associated with effects of structure was observed during the encoding phase only. Also, effects of path-length (Guerard and Tremblay, 2012) and crossings have been shown to be unaffected by manipulations of the amount of rehearsal and by concurrent tapping performed during rehearsal (Parmentier and Andrés, 2006).

It has been proposed that effects of structure in SSR obtained by imposing symmetry, good continuation, and repetition of parts of the sequence in translated positions are to be attributed to the participation of long-term memory and that this could occur at recall (see Kemps, 2001; Rossi-Arnaud et al., 2005, for a discussion of this point). This could be possible. However, it has been shown (De Lillo and Lesk, 2010) that with serial recall tasks presented on touch-screens the effect of clustering and structure occur also in tasks which do not require the reproduction of the sequence at recall (i.e., participants are required to judge if two sequences are the same or not). Therefore, we consider it unlikely that the effects of structure are built at that time or that they are related to the motor plan of the sequence (see also Farrell and Lelievre, 2012, for similar conclusions in relation to temporal grouping).

One likely possibility is that during the encoding phase people start to build a mental image of the search space and the path followed and that the benefits of grouping occur at that stage and can persist during rehearsal. The possible role of mental images in spatial serial recall in the Corsi test makes intuitive sense and has been envisaged by several researchers (see Berch et al., 1998; Guerard and Tremblay, 2012). It is possible that the DLPFC activity that accompanies the benefits of structure in spatial recall (Bor et al., 2003) is related to the activation of mental images of visual patterns conforming to familiar shapes or gestalten. This would be consistent with studies that indicate an involvement of the dorsal part of the prefrontal cortex in tasks requiring mentally imagining visual patterns (Ishai et al., 2000; Mechelli et al., 2004). It is known that grouping can occur in mental images. The extent to which the advantage for structure is mediated by static representations of mentally constructed visual or dynamic shifts of attention rehearsing the pattern remains to be determined.

In summary, there is some inconsistency in the literature in relation to the role of coding strategies, path characteristics and perceptual grouping in explaining beneficial effects of organization. Concepts related to one or the other of these factors are often used interchangeably or considered confounded. The current study clarifies that neither path-characteristics nor perceptual grouping principles can account for all effects of organization in SSR. Importantly, path-length throughout this series of experiments emerged to be the least important factor in explaining the beneficial effects of structure. This is of great interest because path-length is perhaps the path characteristic that is least likely to be related with the efficient encoding of data. Yet, it is the factor that most often is confounded with effects of organization. We have shown that even in navigational tasks where the cost of traveling is exacerbated and produces a large discrepancy in the time taken to observe the sequence at encoding, shorter paths are not always associated with better recall. Interestingly, comparative studies of humans and monkeys on serial recall tasks in small scale tests presented on touch-screens, where path-length and structure were dissociated showed that whereas humans are more sensitive to structure, monkeys are more sensitive to path-length (Fagot and De Lillo, 2011). This may be indicative of a peculiarity of human higher level cognition consisting in an enhanced ability to pick-up structure and use it to efficiently encode information, which could have implications for both the understanding of the evolutionary origins of human cognition and for the refinement of primate models of human memory.

The short-term retention of spatiotemporal structures is ubiquitous in behavior. Yet the cognitive processes supporting it are still poorly understood and comparatively under-investigated in experimental psychology. We believe that disentangling the effects of the efficient use of data structure from effects of path-characteristics in serial recall should open-up new lines of essential research in working memory. These should be aimed at characterizing further the processes responsible for it in different task domains, the extent to which they dissociate in neuropsychological conditions, during lifespan and in animal models of human cognition.

## Author contributions

CD conceived the study, analyzed the data, and wrote the article. MK collected and analyzed data for Experiment 4 and contributed to the write-up of the article. DP collected data of Experiment 3, contributed to the analyses and write-up of that experiment and provided comments on drafts of the article.

### Conflict of interest statement

The authors declare that the research was conducted in the absence of any commercial or financial relationships that could be construed as a potential conflict of interest.

## Footnotes

^1^The set of experiments reported here focused on accuracy rather than response time (RT) and RT analyses were outside the scope of the present study. For detailed analyses of timing in relation to the composition of clustered sequences see De Lillo and Lesk (2010). For traveling time analyses in virtual reality tasks on related topics see Logie et al. (2011) and Trawley et al. (2011).
